# American Academy of Medicine

**Published:** 1896-04

**Authors:** Charles McIntire

**Affiliations:** Secretary, Easton, Pa.


### AMERICAN ACADEMY OF MEDICINE.

PRELIMINARY "PRESS NOTICE" OF TWENTY-FIRST ANNUAL SESSION AT ATLANTA, GEORGIA.

The twenty-first annual session of the American Academy of Medicine will be held in the "Dancing Hall" of the Hotel Aragon, Atlanta, Ga., on Saturday, May 2, and Monday, May 4, 1896.

The proprietors of the Aragon have made special rates for those who attend the meeting; it is confidently expected that the concession for one and one-third fare for the round trip granted to the American Medical Association will be available in time for those who desire to attend the opening session; a very pleasant excursion can be arranged to start from Philadelphia and visit Asheville, North Carolina, the "Land of the Sky," and Chattanooga, en route to Atlanta, if a sufficient number club together for that purpose; or special Pullman cars can be chartered for the exclusive use of those who attend the meeting for the direct route to Atlanta from any rendezvous selected. The secretary invites correspondnce on any of these topics, and also from those who may desire a copy of the completed programme, when it is issued; full information will be promptly given on any of these subjects.

OUTLINE PROGRAM.

The Academy will meet in executive session with closed doors on Saturday, May 2, at 10, A.M. The open session for the reading of papers will begin at about 11 A.M. There will be a recess for lunch from 1 to 2.30 P.M. The "Reunion Session" and annual dinner will be held on Saturday evening. An executive session will be held on Monday morning, after which the special discussion on "Methods of Medical Education," will be the order of the day. The Association of American Medical Colleges, and the Confederation of the State Boards of Medical Examiners and Licensers have accepted the invitation of the Council and will participate in this discussion. The time table for the day and the time for adjournment will be determined by the circumstances.

### PAPERS PROMISED.

NOTE.—No attempt is made to arrange the papers in the order in which they are to be read.

1. "Laboratories and Hospital Work," the President's Address. Henry M. Hurd, M. D., Baltimore, Md.

2. "Colonies for Epileptics." Fredrick Peterson, M. D., New York City.

3. "Insanity in the South." J. T. Searcy, M. D., Tuscaloosa, Ala.

4. "Tuberculosis in Public Institutions." J. W. Babcock, M. D., Columbia, S. C.

5. "Vivisection." George M. Gould, M. D., Philadelphia.

6. Subject not yet given. Woods Hutchinson, M. D., Iowa City, Ia.

7. "The Confusion of Pharmacy, Relating to the Theory and Practice of Medicine," Elmer Lee, M. D., Chicago.

8. A National Board to License for the Practice of Medicine," Henry Leffmann, M. D., Philadelphia.

9. "Report of the Committee to Abstract the Laws Regulating the Practice of Medicine and to Suggest a Model Law:" Perry H. Millard, M. D Chairman, St, Paul, Minn.

10. "Homocide," Paul Bartholow, M D., Philadelphia.

11. "The Sociologic and Scientific Attitude of the Medical Profession." W. J. K. Kline, M. D., Greensburg, Pa.

12. "A Study of Some of the Distinguishing Features of the Homo Medicus," Charles McIntire, M. D., Easton Pa.

### DISCUSSION ON "METHODS OF MEDICAL TEACHING."

This discussion will be opened by a series of ten minute papers, and in the open discussion to follow, each speaker will be limited to five minutes. The opening papers are as follows:

13. "The Preparatory Mental Discipline for the Medical Student." F. H. Gerrish, M. D., Portland, Me.

14. "The Lecture and its Uses." Charles B. Penrose, M. D., Philadelphia, Pa.

15. "Text-book Recitation and its Advantages." De Lancy Rochester, M. D., Buffalo.

16. "Laboratory Methods." V. C. Vaughn, M. D., Ann Arbor, Mich.

17. "Clinical Teaching for Graduates in Diseases of Children." J. Madison Taylor, M. D., Philadelphia.

18. "The Seminary Method." Bayard Holmes, M. D., Chicago.

19. "Examinations." E. L. Holmes, M. D., Chicago.

20. "Students' Medical Societies." Rosewell Park, M. D., Buffalo.

21. "State Examinations," J. McPherson Scott, M., D., (of the Maryland Board of Examiners) Hagerstown, Md.

22. "The Best Method to Teach Anatomy." John B. Roberts, M. D., Philadelphia.

23. "The Best Method to Teach Physiology." Charles D. Smith, M. D., Portland, Me.

24. "The Best Method to Teach 'Practice'." J. C. Wilson, M. D., Philadelphia.

25. "The Best Method to Teach Surgery." J. S. Wright, M. D., Brooklyn.

26. "The Best Method to Teach Obstetrics." J. C. Edgar, M. D., New York City.

27. "The Best Method to Teach State Medicine." George H. Rohe, M. D., Catonsville, Md.

Correspondence is pending about some additional papers; if any more are promised, the titles will appear in the completed programme.

CHARLES MCINTIRE, Secretary,
Easton, Pa.

